# The microbial composition of the initial insult can predict the prognosis of experimental sepsis

**DOI:** 10.1038/s41598-021-02129-x

**Published:** 2021-11-23

**Authors:** Szabolcs Péter Tallósy, Marietta Zita Poles, Attila Rutai, Roland Fejes, László Juhász, Katalin Burián, József Sóki, Andrea Szabó, Mihály Boros, József Kaszaki

**Affiliations:** 1 Institute of Surgical Research, Albert Szent-Györgyi Medical School, University of Szeged, Szeged, Hungary; 2 Department of Medical Microbiology, Albert Szent-Györgyi Health Center and Faculty of Medicine, University of Szeged, Szeged, Hungary

**Keywords:** Experimental models of disease, Outcomes research, Preclinical research, Translational research, Microbial communities

## Abstract

We hypothesized that the composition of sepsis-inducing bacterial flora influences the course of fecal peritonitis in rodents. Saline or fecal suspensions with a standardized dose range of bacterial colony-forming units (CFUs) were injected intraperitoneally into Sprague–Dawley rats. The qualitative composition of the initial inoculum and the ascites was analyzed separately by MALDI-TOF mass spectrometry. Invasive monitoring was conducted in separate anesthetized groups (n = 12–13/group) after 12, 24, 48 and 72 h to determine rat-specific organ failure assessment (ROFA) scores. Death and ROFA scores peaked at 24 h. At this time, 20% mortality occurred in animals receiving a monomicrobial *E. coli* suspension, and ROFA scores were significantly higher in the monomicrobial subgroup than in the polymicrobial one (median 6.5; 5.0–7.0 and 5.0; 4.75–5.0, respectively). ROFA scores dropped after 48 h, accompanied by a steady decrease in ascites CFUs and a shift towards intra-abdominal monomicrobial *E. coli* cultures. Furthermore, we found a relationship between ascites CFUs and the evolving change in ROFA scores throughout the study. Hence, quantitatively identical bacterial loads with mono- or polymicrobial dominance lead to a different degree of sepsis severity and divergent outcomes. Initial and intraperitoneal microbiological testing should be used to improve translational research success.

## Introduction

Experimental models can provide a basis for the development of human therapeutics, but an effective laboratory strategy cannot always be transferred to clinical practice. The most recent Minimum Quality Threshold in Preclinical Sepsis Studies (MQTiPSS) criteria outline the recommended scheme for rodent experimental sepsis^1^ and highlight the importance of consecutive evaluation of established signs of organ failure, similarly to the sequential organ failure assessment (SOFA) scoring systems in humans^2,3^. Nevertheless, findings of preclinical laboratory studies are still difficult to compare^4^, because the magnitude of bacterial load is problematic to standardize and the composition of the intraluminal microbiome at the time of the infection is usually unidentified; the impact of the microbial profile on the progression of events therefore also remains unknown^5^.

The bacterial strains of human or rat stool are broadly representative of the principally polymicrobial flora of the distal colon, and therefore intra-abdominal administration of fecal matter is considered a good rodent model for human peritonitis-linked sepsis. Among the many CLP alternatives^6^, fecal slurries, intra-abdominal injections of fresh or stored solutions of fecal suspensions, can reduce the inherent variance of invasive surgical procedures^7^. Nevertheless, the composition and activity of competing microbial communities may vary greatly even in precisely quantified fecal doses; the results can therefore also be biased when concise qualitative information on the invading microorganisms is lacking^8^. The question of whether a dominant strain or certain strains collectively—a single component or a multicultural microbial community—will determine the initial severity and the natural course of bacterial sepsis remains unresolved.

Based on this knowledge gap, we hypothesized that the unknown qualitative bacterial composition within the fecal mass triggering the insults could be a highly important confounding factor and a decisive descriptor of the course of a septic scenario. Therefore, our primary aim was to investigate the relationship between the bacterial concentration and the qualitative composition of a standardized fecal suspension and the severity of the progressively evolving experimental sepsis as a function of time. To this end, we have used a qualitative, murine-specific sickness scoring system, and we have developed a comprehensive, MQTiPSS-compatible evaluation of organ function parameters to adequately quantify the clinical condition of the septic animals with a rat-specific organ failure assessment (ROFA) scoring system^9^ between 12 and 72 h after induction.

Furthermore, it has been shown that *E. coli* strains alone can lead to fulminant sepsis with high early mortality, while a combination of *E. coli* and other bacterial strains results in the development of a more localized process with intra-abdominal abscess formation^7,10^. Although *E. coli* strains are the most abundant facultative aerobes in the intestines, there are numerous other Gram-negative and Gram-positive pathogens in the microflora that are potentially responsible for gastrointestinal tract-derived sepsis. Fecal infections are generally considered polymicrobial, but the question of whether *E. coli* or other dominant strains of the fecal matter influence the initial severity of sepsis is unresolved. Our second objective was thus to retrospectively analyze the influence of bacterial background on the initial severity and outcome of fecal peritonitis.

Indeed, intra-abdominal sepsis is a dynamic condition involving potentially destructive events when the pro-inflammatory process trespasses the peritoneal cavity and several lines of host defense to eliminate the insult^11^. We therefore hypothesized that changes in intra-abdominal microbial diversity (and the fate of individual members of a microbial community ecosystem) may play a key role in determining sepsis progression since the peritoneal environment and intraperitoneal immunological processes are thought to influence not only the concentration but also the composition of the bacterial culture present in the ascites. Hence, our third objective was to determine the variation of the composition and the change of germ counts of the bacterial populations in the abdominal cavity as a function of time.

Along these lines, we tracked time-dependent changes in the composition of intra-abdominal strains in model experiments with standardized and characterized bacterial load. We have demonstrated that the microbial community structure of the insult may critically influence the course and consequences of bacterial sepsis in laboratory rats. We propose that qualitative, microbiology-based screening should be properly integrated into the design of in vivo sepsis models.

## Results

### Animal well-being and mortality

**Figure 1:**
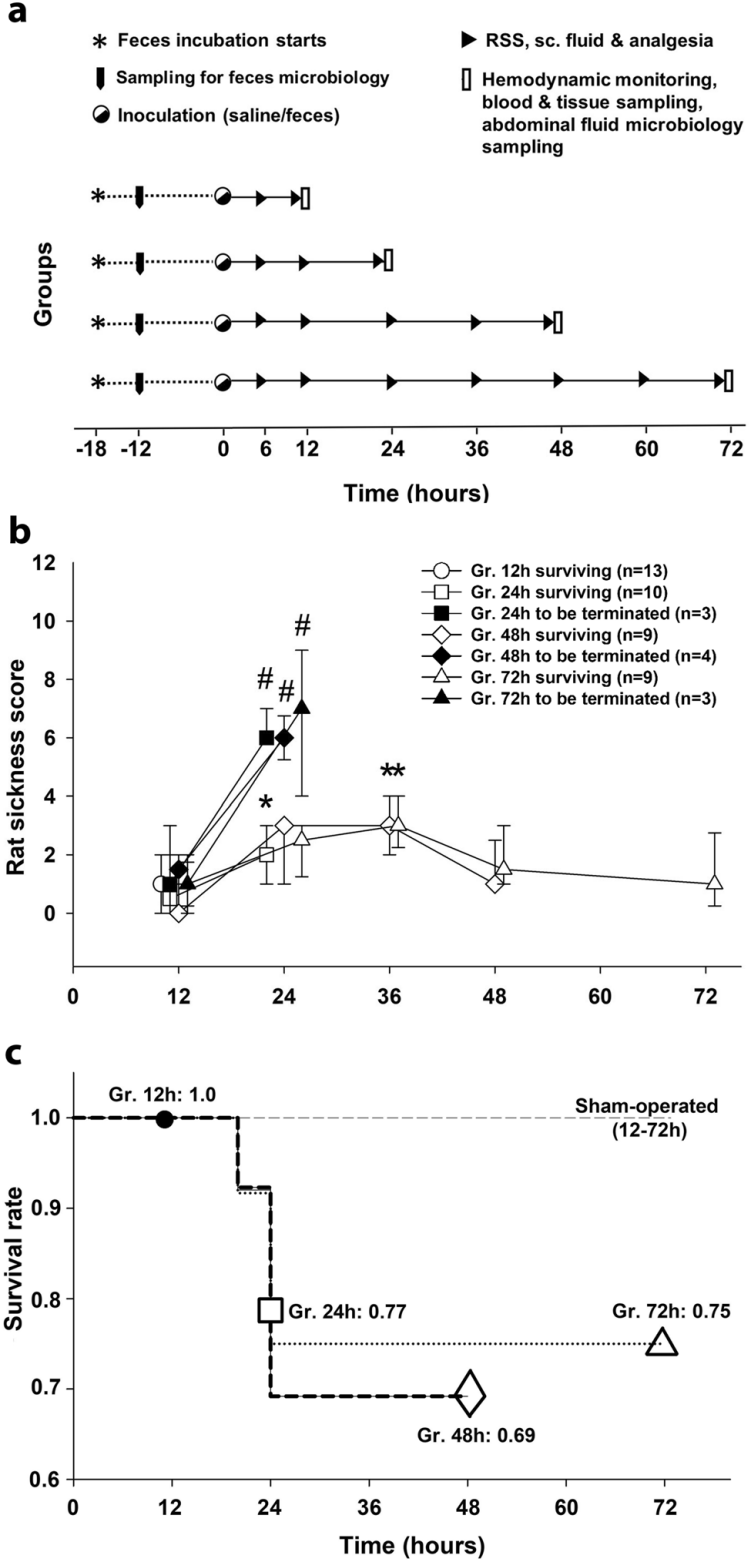
Experimental protocol, rat-specific sickness score (RSS) and mortality at different stages of the sepsis. **(a)** The scheme for the experimental protocol (groups, interventions and assessments). The animals were randomly assigned to sham-operated and septic groups, which were further divided into four independent groups each according to the termination timeline set between 12 and 72 h. Interventions and assessments (including RSS score) are marked with symbols. The animals were injected with an inoculum fitting the 1.02 × 10^6^–5.6 × 10^6^ CFU range. This dosing regimen caused marked RSS changes and organ dysfunction leading to approximately 20% mortality in a pilot study (see Supplementary Material S1). **(b)** Within the cohort of septic animals, RSS score values of surviving animals (open symbols) and those reaching the threshold value of 6 (euthanized subjects; black symbols) are shown separately (*Grs.* groups). Plots demonstrate the median values and the 25th (lower whisker) and 75th (upper whisker) percentiles. Within groups: Friedman test and Dunn’s post-hoc test. *P < 0.05 vs value of non-lethal 12 h sepsis. Between groups: Mann–Whitney U test. ^#^*P* < 0.05 lethal vs non-lethal sepsis at 24 h. **(c)** Kaplan–Meier survival analysis performed on the four sepsis groups (Grs. 12 h, 24 h, 48 h and 72 h) and four sham-operated groups; survival rates are indicated.

Intra-abdominal sepsis was induced by fecal inoculum with known colony forming units (CFUs). Assessments of animal well-being (using a rat-specific, well-being-related sickness score, RSS) and organ dysfunction (using the rat-specific ROFA scoring system, see later) were performed, while the termination of each experiment was scheduled at predetermined time points (12 h, 24 h, 48 h and 72 h) after sepsis induction (Fig. 1a). The rat-specific RSS score did not change significantly at 6 h and 12 h after the septic challenge (Fig. 1b)**,** and there was no mortality in these groups (Fig. 1c). Within nearly 24 h, the condition of some septic animals deteriorated significantly, the RSS values having reached a critical value of 6 (i.e. the threshold for a humane endpoint) and therefore n = 3, n = 4 and n = 3 animals were humanely euthanized in Groups 24 h, 48 h and 72 h, respectively. The number of euthanized animals (the underlying rationale is discussed in the “Materials and methods” section) is included in mortality calculations (Fig. 1c). The general condition of surviving septic animals did not deteriorate between 48 and 72 h, as reflected by RSS scores of less than 2 without further lethality (Fig. 1b).

### Inflammatory biomarkers

**Figure 2:**
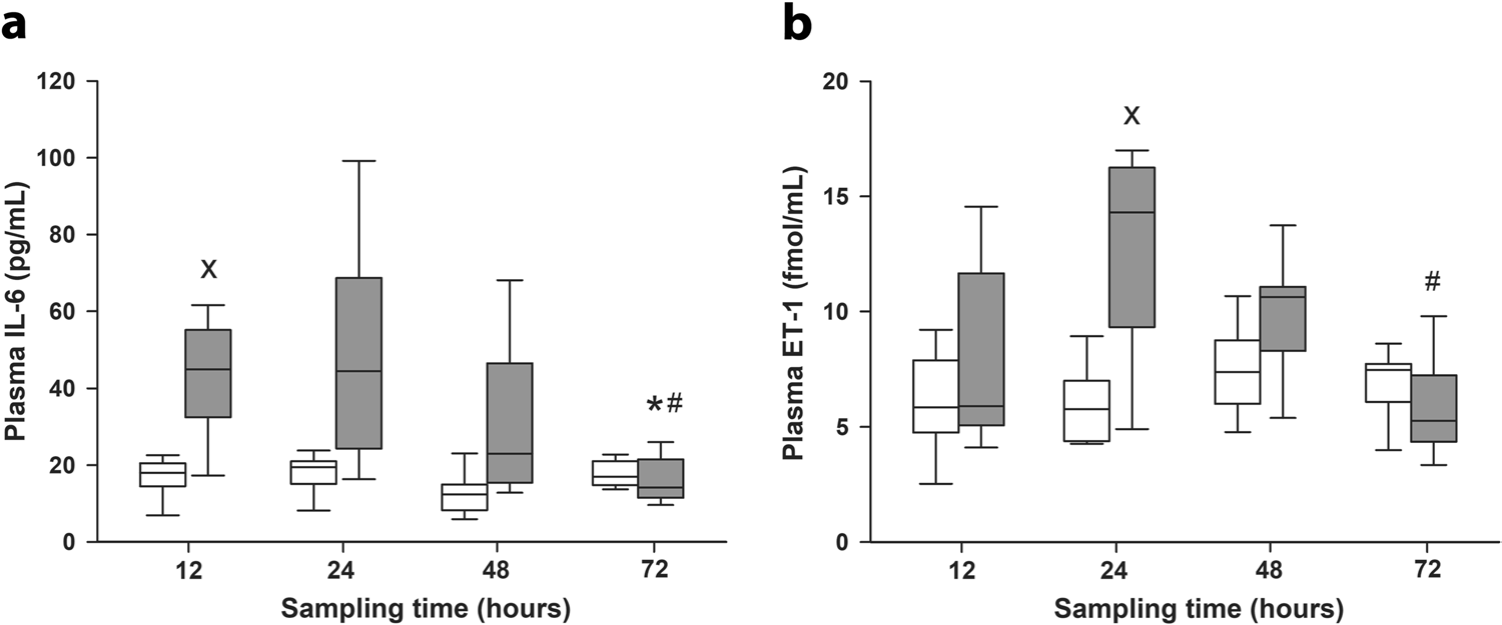
Plasma interleukin 6 (IL-6) **(a)** and endothelin-1 (ET-1) concentrations **(b)** in sham-operated animals (n = 12–13, white boxes) and in the different sepsis groups representing different stages of sepsis (grey boxes). The number of animals used in the various sepsis groups is indicated in Table 1. Plots demonstrate the median (horizontal line in the box) and the 25th (lower whisker) and 75th (upper whisker) percentiles. A comparison between groups was conducted with the Kruskal–Wallis test followed by Dunn’s post-hoc test. ^X^*P* < 0.05 vs sham-operated groups; *P < 0.05 vs 12 h sepsis (between sepsis groups); ^#^*P* < 0.05 vs 24 h sepsis (between sepsis groups).

We determined the plasma levels of interleukin-6 (IL-6; a relatively early biomarker in sepsis) and endothelin-1 (ET-1; a marker of tissue hypoxia) to confirm and characterize the course of pro-inflammatory processes in the animals. Elevated levels of plasma IL-6 were observed 12 h after induction (*P* = 0.018); plasma ET-1 concentration increased significantly at 24 h of sepsis (Fig. 2). Thereafter, levels of both biomarkers returned to those of the sham-operated animals.

### Circulatory and subcellular oxygen dynamics

**Figure 3:**
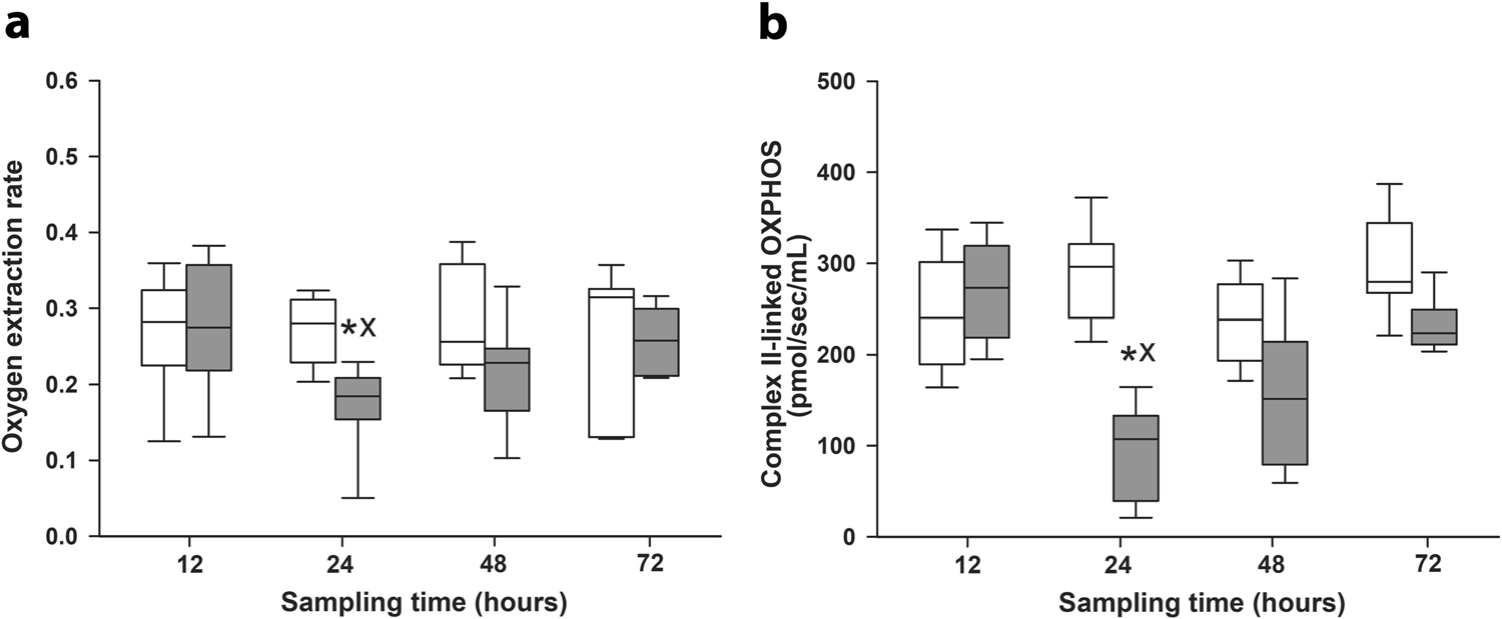
Oxygen extraction rate **(a)** and mitochondrial respiration **(b)** in sham-operated animals (n = 12–13, white boxes) and in the different sepsis groups representing different stages of sepsis (grey boxes). The number of animals used in various sepsis groups is indicated in Table 1. Plots demonstrate the median (horizontal line in the box) and the 25th (lower whisker) and 75th (upper whisker) percentiles. Comparison between groups was conducted with the Kruskal–Wallis test followed by Dunn’s post-hoc test. ^X^*P* < 0.05 vs sham-operated groups; *P < 0.05 vs 12 h sepsis (between sepsis groups); ^#^*P* < 0.05 vs 24 h sepsis (between sepsis groups).

To assess the sepsis-induced tissue hypoperfusion, we determined the systemic oxygen extraction rate (OER) and the efficacy of mitochondrial respiration (from liver biopsies) in sham-operated and septic animals with different observation periods. There was no significant difference in the OER between the sham-operated and septic animals 12 h after septic insult, but the values showed a significant reduction after 24 h of sepsis (Fig. 3a). In sepsis groups with longer observation, less diminished OER values were detected. The Complex II-linked capacity of oxidative phosphorylation—indicative of subcellular oxygen consumption—also shows deterioration in the 24 h septic animals only (*P* < 0.01 vs sham-operated) (Fig. 3b).

### Changes in ROFA score components

**Figure 4:**
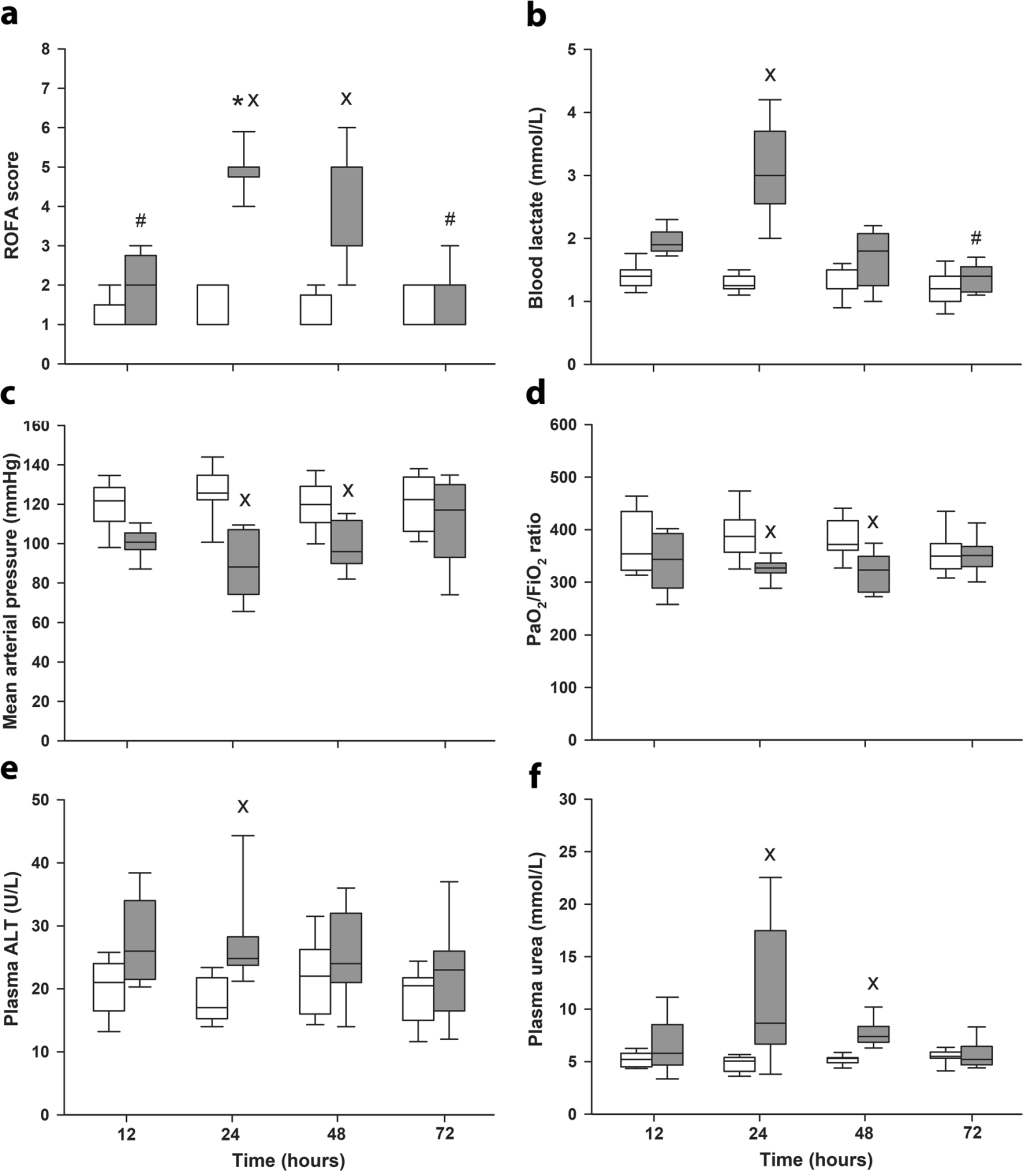
Rat-specific organ failure assessment (ROFA) score and its components in sham-operated animals (n = 12–13, white boxes) and in the different sepsis groups representing different stages of sepsis (grey boxes). Cumulative ROFA score (**a**), plasma lactate levels (**b**), mean arterial pressure (**c**), lung injury (represented by the Carrico index) (**d**), plasma alanine aminotransferase (ALT) (**e**) and plasma urea levels (**f**) are shown. The number of animals used in the various sepsis groups is indicated in Table 1. Comparisons between groups were conducted with the Kruskal–Wallis test followed by Dunn’s post-hoc test. ^X^*P* < 0.05 vs sham-operated groups; *P < 0.05 vs 12 h sepsis (between sepsis groups); ^#^*P* < 0.05 vs 24 h sepsis (between sepsis groups).

The cumulative value of the ROFA score showed significant increases at 24 and 48 h (Fig. 4). Deteriorations in 24 h values were attributable to all parameters examined, whereas similar changes were also present for most parameters at 48 h, except for the significantly not different blood lactate and plasma ALT values. ROFA scores returned to the values of sham-operated animals after 72 h.

## Retrospective analyses

### Microbial features of the fecal inoculum and the ascites

**Figure 5:**
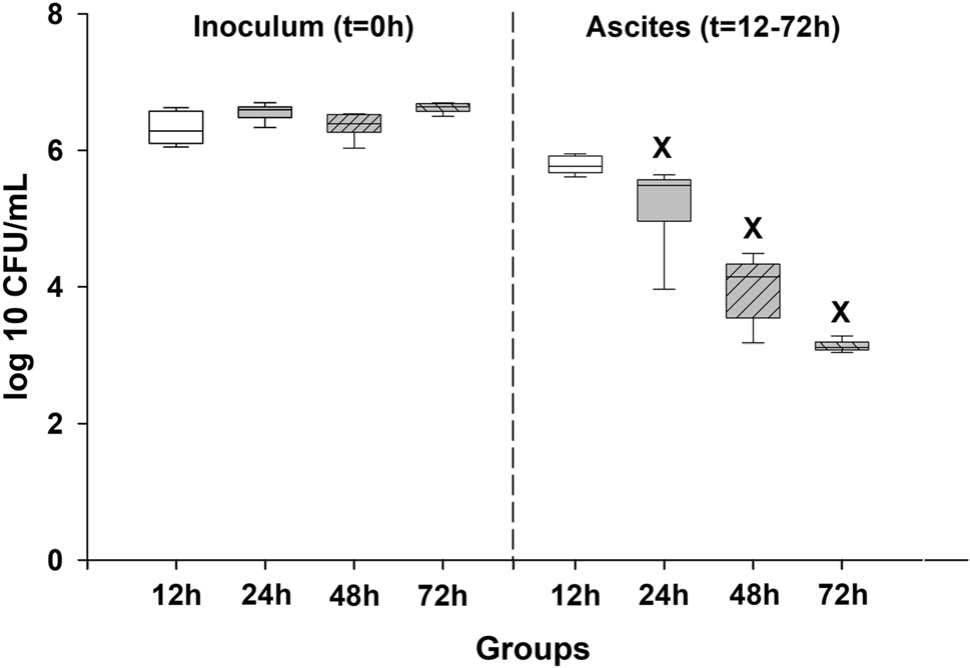
Bacterium concentration in the induction inocula and the abdominal fluids in the different sepsis groups representing different stages of sepsis. The number of animals used in the various sepsis groups is indicated in Table 1. Plots demonstrate the median (horizontal line in the box) and the 25th (lower whisker) and 75th (upper whisker) percentiles. Data were analyzed with two-way analysis of variance (ANOVA) followed by the Holm–Sidak post-hoc test. ^X^*P* < 0.05 vs corresponding inducer inoculum.

In addition to determining the CFUs, analysis of the bacterial pattern (by MALDI-TOF mass spectrometry) was performed from both the sepsis-inducing fecal inocula and the ascites (the latter taken upon termination of the experiments in Grs. 12–72 h). This retrospective qualitative microbiological analysis (48 h after induction) revealed that, despite the statistically similar bacterial doses (Fig. 5), marked qualitative differences (e.g. in bacterial composition and monomicrobial/polymicrobial pattern) existed in the composition of the inocula (Table 1, Supplementary Material S2, Supplementary Table S1). It was proven that only *E. coli* was present in 20% of inocula (nΣ = 10) and all early (24-h) sepsis mortality was attributable to injection with *E. coli* monomicrobial cultures (Table 1). In the sepsis-inducing inocula, three bacterial phyla, including 18 mostly Gram-negative genera, could be detected. Specifically, the majority of taxa belonged to Proteobacteria (49%; e.g. *E. coli*), and the rest were distributed amongst Firmicutes (38%; e.g. Lactobacilli) and Actinobacteria (13%; e.g. *Propionibacterium acnes*) (Supplementary Table S1). The most frequent strains in the inducer inoculum were *E. coli* (in 100% of the samples), followed by *Klebsiella pneumoniae*, Pseudomonas, Bifidobacteria and Lactobacilli.

**Table 1:** Bacterial diversity and sepsis-related mortality.

Groups	Type of sample	Total number of species (bacterial diversity)	Total number of animals available for sampling	Number of animals with polymicrobial culture present (mortality at 24 h)	Number of animals with *E. coli* monomicrobial culture present (mortality at 24 h)
Gr. 12 h	Inoculum	22	13	12 (0/12)	1 (0/1)
	Ascites	13	13	12	1
Gr. 24 h	Inoculum	19	13	10 (0/10)	3 (3/3)
	Ascites	9	10	6	4
Gr. 48 h	Inoculum	22	13	9 (0/9)	4 (4/4)
	Ascites	5	9	5	4
Gr. 72 h	Inoculum	21	12	9 (0/9)	3 (3/3)
	Ascites	2	9	1	8

Bacterial diversity is represented by the total number of bacterial species present in the inoculum and ascites samples in the different sepsis groups (Grs. 12–72 h). Mortality data (ratio of the numbers of animals which had to be humanely euthanized at 24 h after sepsis induction compared to the total number of animals in the given group) are indicated in brackets in the lines for the corresponding inocula.

In the ascites, bacterium concentration decreased by one order of magnitude (*P* < 0.01) (Fig. 5), which was also accompanied by reduced diversity of bacterial strains during the sepsis progression (Table 1). In addition, new species also reached the detection level (Supplementary Table S1). After 72 h of sepsis, only *E. coli* (in 100% of the samples) and *Lactobacillus murinus* (in 17% of the samples) were identified in the ascites.

### Association between bacterial dose of the inducer inoculum and the severity of organ failure

**Figure 6:**
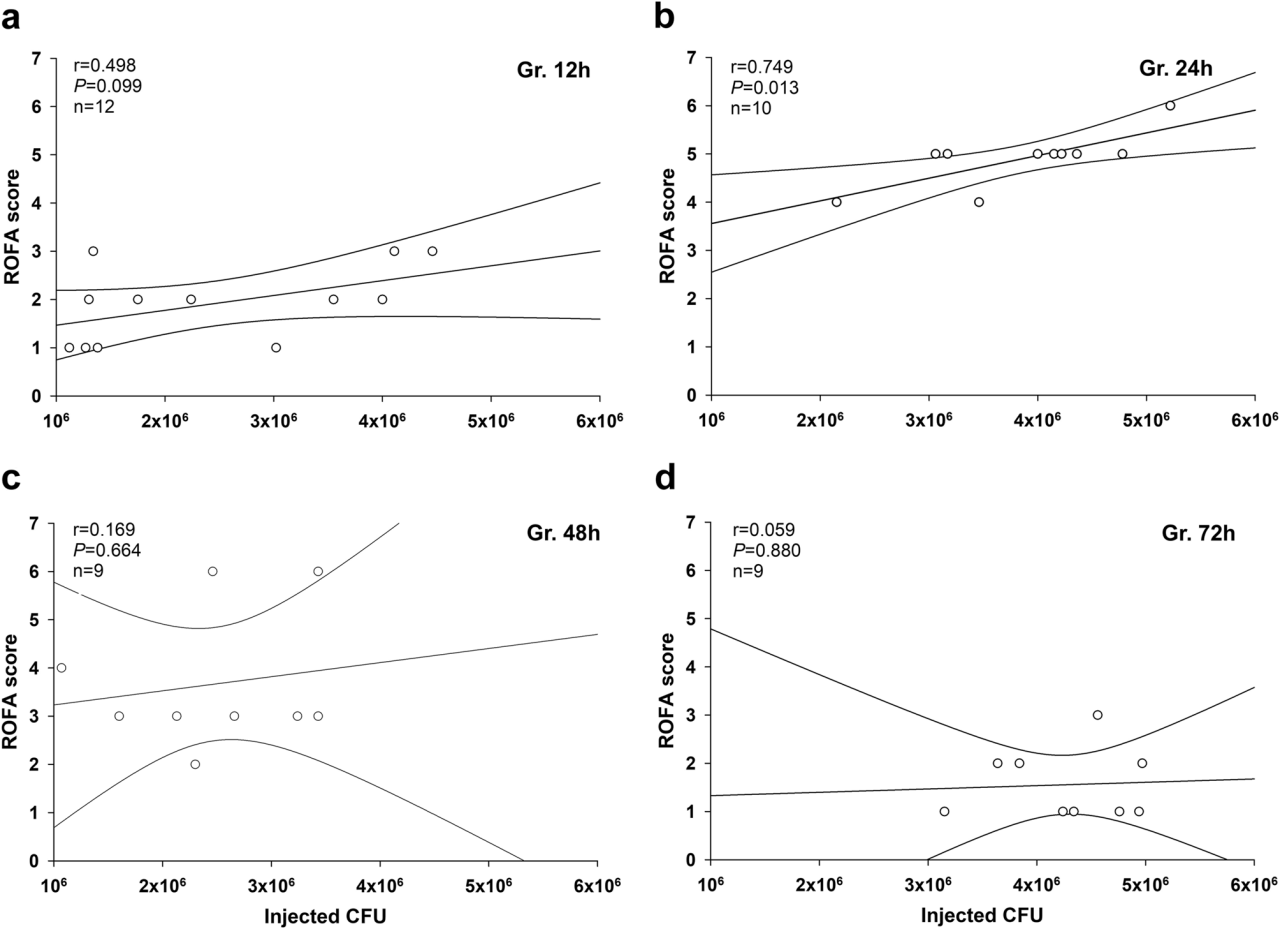
Correlation between CFU values of the inducer inoculum and ROFA scores. Values are shown in the 12 h **(a)**, 24 h **(b)**, 48 h **(c)** and 72 h **(d)** polymicrobial sepsis groups. The number of animals used in the various sepsis groups is indicated in Table 1. Pearson’s product correlation coefficient r values and (null hypothesis-related) *P* values are provided, and regression lines and 95% confidence intervals are indicated.

We also wanted to examine the influence of inoculum CFUs on organ dysfunction (as evidenced by the cumulative ROFA score). The initial CFU and ROFA scores showed a moderate relationship at 12 h (Fig. 6a). However, a significant correlation between these parameters was observed at 24 h of fecal peritonitis (Fig. 6b). No connection between the amount of injected CFU and ROFA score was evidenced at 48 and 72 h (Fig. 6c,d).

### ROFA score components at 24 h after mono- and polymicrobial-type inocula

**Figure 7:**
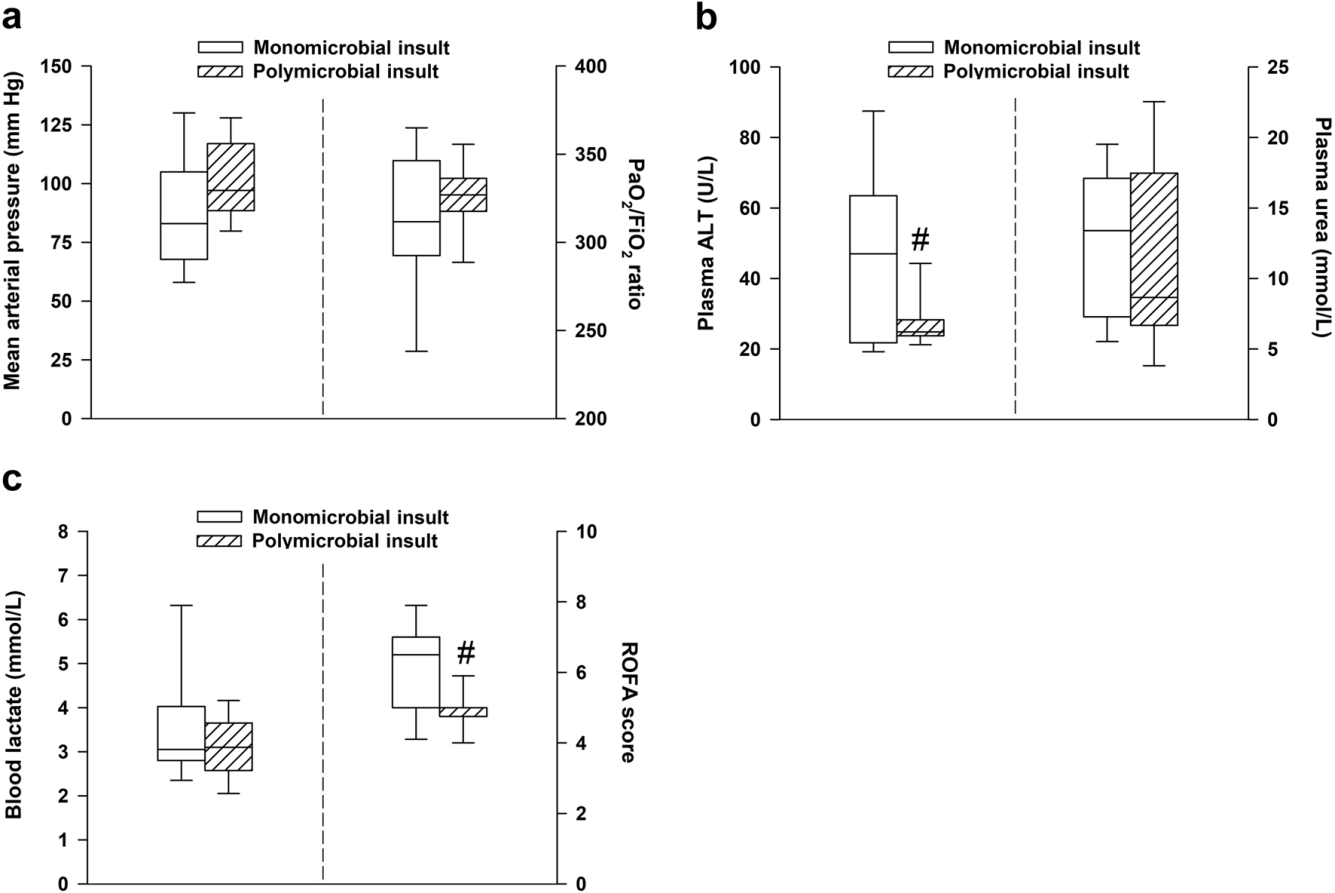
Comparison of rat-specific organ failure assessment (ROFA) parameters in *E. coli* monomicrobial (n = 10) or polymicrobial subgroups (n = 10) at 24 h. **(a)** Mean arterial pressure and lung injury (represented as the Carrico index), **(b)** plasma alanine aminotransferase (ALT) and plasma urea levels, **(c)** blood lactate levels and cumulative ROFA score. Plots demonstrate the median (horizontal line in the box) and the 25th (lower whisker) and 75th (upper whisker) percentiles. Between groups, comparison of ROFA score values was made with the Mann–Whitney U test, whereas ROFA components were compared with the Welch independent samples t-test. ^#^*P* < 0.05 monomicrobial vs polymicrobial septic subgroups.

Since retrospective qualitative microbiological analysis revealed marked differences in the diversity of bacterial strains (also with respect to mono- vs polymicrobial features) in the inoculum, we ran a retrospective subgroup analysis to compare the parameters of ROFA scores for animals that were found to be originally challenged with polymicrobial inoculum (in the 24-h sepsis group; n = 10) and with *E. coli* monomicrobial content in the 24, 48 and 72 h sepsis groups (n = 3, 4 and 3 animals, respectively; n_∑_ = 10). We found significantly higher ALT and ROFA score values in animals injected with *E. coli* monomicrobial inoculum compared to the other cohort (Fig. 7a–c).

**Figure 8:**
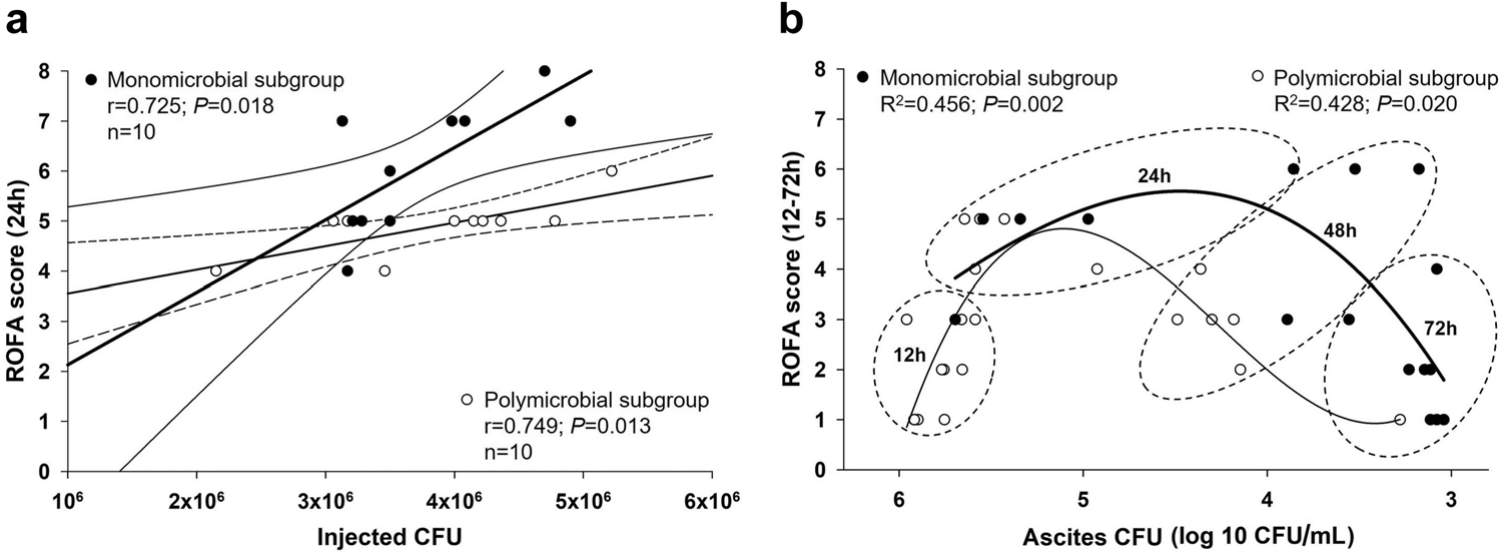
Correlations between inoculum and ascites CFU and ROFA score values in mono- or polymicrobial subgroups. **(a)** Correlations between CFU values for the mono- or polymicrobial inducer inoculum and ROFA scores at 24 h of sepsis. The *E. coli* monomicrobial subgroup (n = 10) is marked with black circles, a thick regression line and a thin line for the 95% confidence interval, whereas the polymicrobial subgroup (n = 10) is indicated with open circles, a thin regression line and a dashed line for the 95% confidence interval. Pearson’s product correlation coefficient r values, (null hypothesis-related) *P* values and numbers of animals involved in the subgroup analysis are provided. **(b)** Relationship between the log 10 CFU values for ascites and ROFA score values in all septic groups. The number of animals in the various sepsis groups is indicated in Table 1. Evolving predominance of ascites monomicrobial (black circles) vs polymicrobial (open circles) content and reduced CFUs in ascites over time. Polynomial regression curves of the polymicrobial and monomicrobial ascites samples are marked with a thin and a thick line, respectively. Third-order polynomial curve fit values (R^2^ and *P*) are indicated. Data belonging to different stages of sepsis are illustrated with dashed ellipses.

Bearing in mind the negligible quantitative differences in inocula, but the remarkably large differences in their microbiological diversity, we also retrospectively assessed how simultaneous consideration of these features influenced organ dysfunction at 24 h of sepsis. According to results from the subgroup analysis, the relationship between CFU values and ROFA scores showed a similar correlation in both the *E. coli* monomicrobial and the polymicrobial subgroups, but the slope of the regression line of the monomicrobial subgroup was greater than that of the polymicrobial subgroup (Fig. 8a).

Furthermore, the ROFA score also displayed a correlation with ascites CFU values. A significant non-linear relationship was identified between the ascites CFU and the ROFA score (3rd order polynomial regression) in polymicrobial (*P* = 0.002) or monomicrobial (*P* = 0.02) subgroups during the 12–72 h sepsis period with a shifting of the bacterial composition towards *E. coli* dominance over time (Fig. 8b).

## Discussion

Our aim was to investigate the microbiological characteristics of a typical rodent model of intra-abdominal sepsis. Except for antibiotics, the study followed the recommendations in the MQTiPSS guidelines; analgesia, standard fluid therapy and strictly defined scoring systems were used to determine the progression of events^2^. The inflammatory immune response was manifested in distinctive, time-dependent increases in plasma IL-6^12^ and ET-1^13^ levels, and the numerical parameters of cardiovascular, pulmonary, kidney and liver functions—all included in the human SOFA scoring system—established the onset, peak and resolution. The functional changes of organ systems were accompanied by signs of subcellular metabolic dysfunction, increased blood lactate levels and reduced Complex II-linked capacity of oxidative phosphorylation of liver mitochondria. In this experimental system, the relationship between the quantified responses and the matching microbiological profile was determined sequentially, starting from the fecal induction to 72 h samples from the abdominal cavity.

A number of preclinical animal models of human sepsis with various advantages and disadvantages have already been presented to the scientific community. In general terms, CLP is considered the most effective investigative tool; nevertheless, precise control of intestinal leakage is usually impossible. Fecal inductions with standardized CFU concentration ranges are considered appropriate alternatives^14^, and the bacterial load can be quantitatively defined. In our hands, 1.02 × 10^6^–5.6 × 10^6^ CFUs correlated well with the onset time of severe reactions 24 h later. This CFU range resulted in approx. 20% summed mortality, broadly reproducing the 17–29% mortality rate of human intra-abdominal sepsis^15^. Here it should be noted that 24 h for rats corresponds to a time interval of approx. 21 days in humans, due to the often overlooked 1:21 conversion ratio of the rat-human age correlation^16^. The changes in signs and symptoms were followed further up to 72 h; visible and measurable parameters were monitored and recorded sequentially, corresponding again to clinical practice. This approach demonstrated the resolution phase; the compensatory mechanisms led to recovery in the surviving cohort of animals between 24 and 72 h.

It should be noted that the flora of fecal peritonitis is generally considered polymicrobial, but identification of endogenous or exogenous microbial sources is usually lacking in the design of animal model strategies. *E. coli* showed the highest frequency among the sepsis-causing bacteria we isolated (followed by *Klebsiella*, *Pseudomonas* and *Acinetobacter*), which is consistent with clinical experience^17–19^. However, sepsis with a Gram-positive source in rodents is relatively rare, in contrast to the frequent occurrence of *Staphylococcus aureus* in human sepsis^19^.

Due to the time burden and fastidious identification of fecal pathogens, there are likewise no established qualitative criteria in CFU values either. Here we have demonstrated the paramount importance of identifying inducer strains because approx. 20% of the fecal solutions with a presumed polymicrobial habitat proved to be monomicrobial (with only *E. coli* present). Moreover, the monomicrobial induction was attributed exclusively to the 24 h mortality, and the general condition of the hosts was more severely impaired in these cases. This finding supports the view that *E. coli* may be responsible for early mortality in intra-abdominal sepsis^20^ but partially contradicts other results, where polymicrobial infections had a higher mortality rate^21^.

Here it should be underlined that a shift from polymicrobial to monomicrobial content had begun during the preparation of the uniformly prepared and mixed stool samples, and the transition was then tracked in the animals’ bodies. Another distinctive feature of the process was the Lactobacillus and Bifidobacteria cultures detected in the ascites perhaps as a compensatory sign of the host’s bacterial defense mechanism^22^. These processes may also explain the absence of mortality in the polymicrobial sepsis group in our experiments.

We followed the dynamics of the transition of bacterial strains in the surviving animals, and the concentration and diversity of the polymicrobial cultures in the ascites decreased after 24 h and the most frequent strain was again *E. coli*^23–25^. However, new strains (e.g. *Neissera subflavia*) were also detected over time, consistent with the number of bacteria in secondary peritonitis. Finally, the majority of the strains disappeared from the ascites, and only *E. coli* and *Lactobacillus murinus* were detected with reduced concentrations in the 72 h samples. Again, these findings indirectly support the hypothesis that Lactobacilli suppress the growth of opportunistic pathogens colonizing in the peritoneum^22^.

To our knowledge, this is the first study to demonstrate the dominant appearance of *E. coli* monomicrobial cultures in association with early mortality in experimental sepsis. A natural selection that is amplified under favorable conditions is the most likely explanation for the process^26,27^, leading to microbial fitness and the “survival of the meanest” phenomenon. It should be noted that the results do not contradict MQTiPSS recommendations^2^, as polymicrobial induction reflects the development of human sepsis better compared to monomicrobial inoculation^14^; however, besides dose responses, there is an obvious need for additional microbial tests to identify the dynamics of microbial changes.

### Limitations

Our cross-sectional characterization reflected the most important features of the clinical disease, but extended monitoring may add further information. Further, the microbial identification time can be improved with novel methods, such as broad-range PCR amplification with High-Resolution Melt Analysis^28^. Only a population of healthy animals was evaluated without age and gender differences, and a number of other variables were not collected which have been demonstrated to influence bacterial reactions, such as diet composition. It should be added that antibiotics affect mitochondrial functions and microbial reactions as well; this confounding option was therefore purposefully omitted from the protocol.

## Conclusions

The colonizing bacteria in the peritoneal sac significantly influence the outcome, so strict microbiological analysis of the qualitative properties and a reliable separation of possible variations—Gram-negative, Gram-positive, mono- or polymicrobial induction types—must be controlled to properly model bacterial sepsis. A similar phenomenon may conceivably occur in human conditions, but we did not want to make assumptions. Nevertheless, enhanced understanding of the relative emergence of dominant strains or species in a mixed culture and the competitive intraperitoneal bacterial responses is likely to improve translational research success. Qualitative microbial changes are of pivotal impact in the pathophysiology, the unknown microbiological profile may define/restrict the usability of rodent sepsis models.

## Materials and methods

### Animals

Male Sprague–Dawley rats (380 ± 30 g bw) were used with adherence to NIH guidelines and EU directive 2010/63/EU for the protection of animals used for scientific purposes, and the study was approved by the national competent authority of Hungary (ATET; V/175/2018). The animals were housed in plastic cages (21–23 ℃) with a 12/12 h dark–light cycle and access to standard rodent food and water ad libitum. The study design and the presentation of the data are in accordance with the MQTiPSS recommendations and with the Animal Research: Reporting of In Vivo Experiments guidelines (https://arriveguidelines.org/).

### Preparation of the sepsis-inducing fecal inoculum

Our aim was to induce peritoneal sepsis with injections of standardized bacterial counts within a defined range, but without limiting the variability in the microbiome composition. Therefore, we did not use a single stock of feces; instead, we prepared fresh fecal inocula on a daily basis (using a standardized method, see below) and injected a maximum of four rats per day according to a predefined randomization protocol (Fig. 1a). This protocol was repeated several times consecutively throughout the duration of the study.

Fresh feces (~ 4 g) was randomly collected from age and body weight-matched healthy rats (n = 4–5) 18 h before the scheduled intra-abdominal injections. The fecal mass was mixed with 6 mL saline in sterile 10 mL Falcon tubes, vortexed and incubated for 6 h at 37 ℃. After a 3:1 dilution, the suspension was filtered to remove the pellet. For microbiological analysis, 0.1 mL samples were taken from the suspension to determine the number of CFUs and identify the strains (qualitative analyses; see later), and the rest of the filtrate was stored at 4 ℃ for 12 h (the typical time required to determine CFUs). In prior studies, we proved that a 6-h incubation period and a 12-h cold storage had no significant effect on the quantitative and qualitative characteristics of these fecal suspensions (Supplementary Fig. S1, Supplementary Table S2).

### Sepsis induction, and experimental setup and groups

The optimal germ count required for reproducible sepsis induction (the relationship between CFUs and mortality rate) was determined in another 24 h pilot study (n = 12, Supplementary Material S1). Based on these in vivo data, the filtered inoculum was injected intraperitoneally (ip.) using a 21G needle in a volume of 5 mL/kg at a dose range of 1.02 × 10^6^–5.6 × 10^6^ CFU. Rats in the sham-operated groups received saline in the same volume. Sample size estimation was performed assuming approx. 20% mortality after 24 h. If the presumed true hazard ratio of septic subjects relative to controls is 0.2 with a power of 1 − β = 0.9 and the Type I error probability is α = 0.05, the inclusion of 12 septic and 12 control animals was recommended in each selected time point. In line with the sample size estimation, the animals were randomly assigned to sham-operated (n_∑_ = 49) and septic groups (n_∑_ = 51), which were randomly further divided into four independent groups each (sham-operated: n_12h_ = 13, n_24h_ = 12, n_48h_ = 12, n_72h_ = 12; septic: n_12h_ = 13, n_24h_ = 13, n_48h_ = 13, n_72h_ = 12) according to a termination timeline set between 12 and 72 h (Fig. 1a).

### Assessments and measurements

#### Quantitative microbiological analysis

The number of CFUs was determined with the standard pour-plate count method^29^ and converted to the cell number per milliliter of the original inocula (CFU/mL). Briefly, a 0.1 mL sample of the final suspension was diluted (1:10), and the dilutions were spread with a glass rod on solid media (Tryptic Soy Agar; Merck, Darmstadt, Germany) under sterile conditions, incubated for 12 h at 37 ℃. Thereafter, the bacterium concentration was assessed by averaging the counted colonies multiplied by the appropriate dilution factor.

#### Qualitative microbiological analysis

The bacterial composition of the inoculum was analyzed with species-selective media for the most frequent species and by MALDI-TOF mass spectrometry (MS; Bruker Daltonics, Germany). A 0.1 mL of the suspension was spread on Mueller–Hinton solid media (Bio-Rad, Budapest, Hungary) to identify the aerobe strains. After a 12 h incubation period at 37 °C, colonies from the mixed culture were isolated to form pure bacterium cultures. Anaerobe strains were inoculated on Columbia agar base (Oxoid, Budapest, Hungary) supplemented with 5% (v/v) bovine blood, hemin (1 mg/ mL) and vitamin K1 (5 mg/mL) for 48 h at 37 °C; anaerobes were incubated in an anaerobic chamber (Bactron, Sheldon Manufacturing, Cornelius, Oregon, US).

Sample preparation for MALDI-TOF MS measurement was performed as described earlier^30^. In brief, the spectra from the microbiological samples were acquired using the Microflex LT system (Bruker Daltonik, Bremen, Germany) and analyzed with MALDI BIOTYPER 3.3 (Bruker Daltonik, Bremen, Germany) software. MALDI-TOF MS analysis was performed in triplicate, with parallel tests performed on the same target plate. The mass spectrometric identification of the microorganisms was confirmed if the score for at least one of three spots was above 2.0 (species level) and above 1.7 (genus level)^31,32^. The results of the bacterium composition were always available 48 h after the end of the preparation procedure of the inoculum.

#### Animal well-being

The general condition of the animals was evaluated at 6 h after the ip. injections and every 12 h thereafter using a modified 0–9 point rat-specific RSS scoring system^33^, where a cumulative value above 6 was considered a humane endpoint for euthanasia (Supplementary Table S3). At time points of sickness assessment, the animals received 10 mL/kg crystalloid solution sc. (Ringerfundin, B. Braun, Hungary) to avoid dehydration and 15 µg/kg buprenorphine sc. (Bupaq, Merck, USA) to maintain analgesia^2^.

#### Hemodynamic measurements: blood and tissue sampling

At the predetermined timeline (12 h, 24 h, 48 h or 72 h after induction), the animals were anesthetized with a mixture of ketamine (45.7 mg/kg ip.) and xylazine (9.14 mg/kg ip.) and placed on a heating pad to maintain body temperature at 37 ℃. Tracheostomy was performed, with the right jugular vein cannulated for fluid infusion (10 mL/kg/h Ringerfundin) and continuous anesthesia (12.2 mg/kg ketamine, 2.52 mg/kg xylazine and 0.612 mg/kg diazepam iv.). The left carotid artery was cannulated to monitor mean arterial blood pressure and heart rate (Cardiosys 1.4, Experimetria Ltd., Budapest, Hungary). After an approx. 30-min surgical preparation and 15-min stabilization, hemodynamic monitoring was performed for 30 min. Arterial and venous blood samples were collected for blood gas (Cobas b123, Roche Ltd., Basel, Switzerland) and blood lactate analysis (Accutrend Plus, Roche, Hungary). Based on a standard formula (SaO_2_ − SvO_2_)/SaO_2_), simplified OER was calculated from arterial (SaO_2_) and venous oxygen saturations (SvO_2_). Lung function was determined by calculating the PaO_2_/FiO_2_ ratio (Carrico index) from partial arterial oxygen pressure (PaO_2_) and FiO_2_ (which was 0.21).

After recording for 30 min, a sterile midline laparotomy was performed and a 0.1 mL fluid sample was taken from the abdominal cavity for microbiological analysis^30^. Next, a tissue biopsy was taken from the left lateral lobe of the liver in cold phosphate-buffered saline to measure mitochondrial functions (see later), and blood samples were collected from the inferior caval vein (see later) using a sterile technique. After sampling, the rats were euthanized with an overdose of ketamine (120 mg/kg).

#### Sequential organ failure assessments

The severity of organ dysfunction was determined with the ROFA scoring system^2,9^, which simultaneously considers not only cardiovascular, respiratory, hepatic and renal damage/dysfunction, but also blood lactate levels. Based on this scoring, sepsis was defined as a cumulative ROFA score above 2 (Table 2).

**Table 2:** Threshold values of the components of rat-specific organ failure assessment (ROFA) scoring system.

ROFA score	Plasma lactate (mmol/L)	MAP (mm Hg)	PaO_2_/FiO_2_ ratio	Plasma ALT (U/L)	Plasma urea (mmol/L)
0	< 1.64	> 75	> 400	< 17.5	< 7.5
1	1.64 < 3	65 < 75	300 < 400	17.5–30.2	7.5–21
2	3 < 4	55 < 65	200 < 300	> 30.2	> 21
3	4 < 5	< 55	100 < 200	–	–
4	> 5	–	< 100	–	–

Sepsis was defined as cumulative ROFA score above 2.

*MAP* mean arterial pressure, *ALT* alanine aminotransferase.

#### Measurements of inflammatory and organ function-related markers

Blood samples were collected from the inferior vena cava into pre-cooled, EDTA-coated tubes, centrifuged (1200×*g* at 4 °C for 10 min) and stored at − 70 °C. Plasma IL-6 and ET-1 levels were determined with standard ELISA kits (Cusabio Biotechnology Ltd., Wuhan, China, and Biomedica Ltd., Vienna, Austria, respectively). Kidney injury was determined from plasma urea level, whereas liver dysfunction was assessed by measuring the plasma alanine aminotransferase (ALT) level, using a Roche/Hitachi 917 analyzer (F. Hoffmann-La Roche AG, Switzerland). All analyses were performed on coded samples in a blinded fashion.

#### Examination of mitochondrial function

Mitochondrial respiration from liver homogenates was determined using high-resolution FluoRespirometry (Oxygraph-2k, Oroboros, Innsbruck, Austria) as described previously^9^. In brief, after Complex I (CI) inhibition by rotenone (0.5 µmol/L, Merck, USA), samples were stimulated with Complex II (CII)-specific substrate (succinate, 10 mM, Merck, USA) for measuring LEAK respiration (LEAK_S_). CII-linked oxidative phosphorylation (OXPHOS CII) was determined at a saturation concentration of ADP (5 mmol/L, Merck, USA). ATP synthase was inhibited by oligomycin (2.5 μmol/L) to assess LEAK respiration in a non-phosphorylating state (LEAK_Omy_). At the end of the protocol, mitochondrial respiration was blocked by Complex III inhibitor Antimycin A (2.5 μmol/L, Merck, USA) to evaluate residual oxygen consumption (ROX). Mitochondrial oxygen consumption (volume-specific flux, *JO*_2_) was expressed in pmol/s/mL.

### Statistical analysis

Data were evaluated with the SigmaStat 12.5 software package (Systat Software, San Jose, CA) or the IBM SPSS 26 software (IBM Corp., Armonk, NY). Survival was analyzed and plotted using the Kaplan–Meier method. Based on data distribution (Shapiro–Wilk test), non-parametric (Mann–Whitney, Kruskal–Wallis or Friedman analysis of variance on ranks tests with the Dunn’s post-h test) or parametric methods (t-test and two-way analysis of variance followed by the Holm–Sidak test) were used. Data were displayed as median values and interquartile ranges of the 75th and 25th percentiles, with *P* < 0.05 being considered significant. The Pearson’s method was used for analysis of linear correlation (correlation coefficient (r), regression lines and 95% confidence intervals were indicated), whereas the non-linear relationship was examined with the cubic polynomial regression analysis (trend line, R^2^ and *P*-values were provided).

## Supplementary Information

Supplementary Information.

## Data Availability

All data generated or analyzed during this study are included in this published article (and its Supplementary Information files).
