# Supplementary material for: The microbial composition of the initial insult can predict the prognosis of experimental sepsis
Source: Sci Rep. 2021 Nov 23;11:22772. doi: 10.1038/s41598-021-02129-x (PMC8611025; doi:10.1038/s41598-021-02129-x)
Supplement: Supplementary file 1 — Supplementary Information. [file 41598_2021_2129_MOESM1_ESM.docx]

**Supplementary Material**

**The microbial composition of the initial insult can predict the prognosis of experimental sepsis**

Szabolcs Péter Tallósy PhD^1^, Marietta Zita Poles PhD^1^, Attila Rutai^1^, Roland Fejes^1^, László Juhász PhD^1^, Katalin Burián MD, PhD^2^, József Sóki PhD^2^, Andrea Szabó MD, PhD^1^, Mihály Boros MD, DSc^1^, József Kaszaki PhD^1🖂^

^1^Institute of Surgical Research, Albert Szent-Györgyi Medical School, University of Szeged

^2^Department of Medical Microbiology, Albert Szent-Györgyi Health Center and Faculty of Medicine, University of Szeged

**Contents**

[Supplementary Material S1. Determination of the optimal germ count and the effects of filtration for the sepsis-inducing fecal inoculum 2](#_Toc80713074)

[Supplementary Table S1. Bacterium composition in the fecal inoculum and the ascites 3](#_Toc80713075)

[Supplementary Material S2. Optimization of the sepsis-inducing fecal inoculum – pilot *in vitro* microbiological studies 4](#_Toc80713076)

[S2.1. Effects of incubation time on microbial diversity 4](#_Toc80713077)

[S2.2. The effects of 12 h storage time at 4 °C on changes in germ count and composition of the induction inoculum 5](#_Toc80713078)

[Supplementary Table S3. Assessment scheme of the condition of animals with rat sickness scores. 6](#_Toc80713079)

# Supplementary Material S1. Determination of the optimal germ count and the effects of filtration for the sepsis-inducing fecal inoculum

A 24 h *in vivo* pilot study was run before the full-scale experiments to test the microbiological features of the induction inoculum which may influence the progression of sepsis. A separate group of animals (n = 12) received different doses of the fecal inducer suspension (3–6 mL/kg) with or without filtration to test the relationship between germ count and mortality rate. A rat-specific sickness scoring system was used to assess the general condition and the need for a humane endpoint.

There was no mortality below the 1.02×10^6^ CFU, and no significant changes occurred in the sickness score for 24 h. However, microbial concentration above 5.6×10^6^ CFU resulted in a high mortality rate (> 90%) in the first 12–16 h (data not shown). Therefore, bacterial content between 1.02×10^6^ and 5.6×10^6^ CFU was administered to investigate sepsis-associated organ dysfunctions and changes in the study groups.

In addition, the influence of filtration was also examined in the context of mortality. It was found that the inducer suspension must be freed from the larger solid plaque to achieve appropriate and reproducible septic syndrome. Filtration significantly reduces the random variability of fecal flocculants providing an adhesion surface for living microorganisms; the homogeneity of the suspension therefore leads to homogeneity in physiological changes as well.

# Supplementary Table S1. Bacterium composition in the fecal inoculum and the ascites

Bacterial composition of fecal inoculum (Inoc.) and ascites (Asc.) expressed as an incidence of the actual strain per total number of samples (%) in the different sepsis groups.

| **Gram reaction** | **Family** | **Species** | **Groups** | | | | | | | | |
| --- | --- | --- | --- | --- | --- | --- | --- | --- | --- | --- | --- |
|  |  |  | **Group 12 h** | | **Group 24 h** | | **Group 48 h** | | **Group 72 h** | | |
|  |  |  | **Inoc.** | **Asc.** | **Inoc.** | **Asc.** | **Inoc.** | **Asc.** | **Inoc.** | **Asc.** |  |
|  |  |  | **(n=13)** | **(n=13)** | **(n=13)** | **(n=10)** | **(n=13)** | **(n=9)** | **(n=12)** | **(n=9)** |  |
| **Gram-negative** | Aeromonadaceae | *Aeromonas caviae* | 33 | 0 | 42 | 0 | 8 | 0 | 25 | 0 |  |
|  | Burkholderiaceae | *Ralstonia pickettii* | 0 | 32 | 0 | 33 | 0 | 0 | 0 | 0 |  |
|  | Caulobacteraceae | *Brevundimonas diminuta* | 33 | 0 | 33 | 0 | 17 | 0 | 17 | 0 |  |
|  | Enterobacteriaceae | *Escherichia coli* | 100 | 100 | 100 | 100 | 100 | 100 | 100 | 100 |  |
|  | Enterobacteriaceae | *Klebsiella pneumoniae* | 92 | 0 | 75 | 0 | 65 | 0 | 72 | 0 |  |
|  | Moraxellaceae | *Acinetobacter radioresistens* | 92 | 50 | 71 | 33 | 68 | 0 | 67 | 0 |  |
|  | Moraxellaceae | *Acinetobacter sp.* | 33 | 0 | 42 | 0 | 8 | 0 | 33 | 0 |  |
|  | Neisseriaceae | *Neisseria subflava* | 0 | 67 | 0 | 29 | 0 | 33 | 0 | 0 |  |
|  | Pseudomonadaceae | *Pseudomonas aeruginosa* | 90 | 31 | 73 | 21 | 33 | 0 | 25 | 0 |  |
|  | Pseudomonadaceae | *Pseudomonas stutzeri* | 33 | 0 | 25 | 0 | 33 | 0 | 67 | 0 |  |
|  | Pseudomonadaceae | *Pseudomonas mucidolens* | 33 | 0 | 42 | 0 | 8 | 0 | 0 | 0 |  |
|  | Veillonellaceae | *Veilonella criceti* | 0 | 0 | 0 | 0 | 33 | 0 | 25 | 0 |  |
|  | Xanthomonadaceae | *Stenotrophomonas maltophilia* | 67 | 30 | 75 | 33 | 0 | 0 | 8 | 0 |  |
| **Gram-positive** | Bifidobacteriaceae | *Bifidobacterium animalis* | 33 | 79 | 25 | 61 | 33 | 33 | 67 | 0 |  |
|  | Enterococcaceae | *Enterococcus ratti* | 17 | 0 | 4 | 0 | 8 | 0 | 54 | 0 |  |
|  | Enterococcaceae | *Enterococcus faecium* | 8 | 67 | 8 | 33 | 42 | 0 | 33 | 0 |  |
|  | Enterococcaceae | *Enterococcus gallinarum* | 0 | 0 | 0 | 0 | 33 | 0 | 33 | 0 |  |
|  | Lactobacillaceae | *Lactobacillus intestinalis* | 90 | 0 | 68 | 0 | 67 | 0 | 67 | 0 |  |
|  | Lactobacillaceae | *Lactobacillus murinus* | 33 | 83 | 33 | 62 | 50 | 60 | 68 | 17 |  |
|  | Lactobacillaceae | *Lactobacillus johnsonii* | 8 | 0 | 0 | 0 | 67 | 0 | 33 | 0 |  |
|  | Lactobacillaceae | *Lactobacillus mali* | 8 | 0 | 0 | 0 | 33 | 0 | 33 | 0 |  |
|  | Lactobacillaceae | *Lactobacillus garvieae* | 0 | 0 | 0 | 0 | 0 | 0 | 33 | 0 |  |
|  | Micrococcaceae | *Micrococcus luteus* | 33 | 83 | 0 | 67 | 33 | 48 | 17 | 0 |  |
|  | Propionibacteriaceae | *Proprionibacterium acnes* | 8 | 0 | 4 | 0 | 17 | 0 | 0 | 0 |  |
|  | Staphylococcaceae | *Staphylococcus aureus* | 50 | 30 | 75 | 8 | 25 | 42 | 17 | 0 |  |
|  | Staphylococcaceae | *Staphylococcus hominis* | 67 | 0 | 58 | 0 | 8 | 0 | 0 | 0 |  |
|  | Streptococcaceae | *Streptococcus hyointestinalis* | 67 | 0 | 58 | 0 | 0 | 0 | 0 | 0 |  |

# Supplementary Material S2. Optimization of the sepsis-inducing fecal inoculum – pilot *in vitro* microbiological studies

## S2.1. Effects of incubation time on microbial diversity

We investigated the effects of 10 h of incubation time on the microbial diversity of rat feces samples. Fresh feces (4 g) was collected from 4–5 randomly chosen rats in the animal facility. The fecal mass was mixed with 6 mL saline in sterile Falcon tubes, homogenized and incubated for 10 h at 37 °C. The diversity of bacterial strains in the suspension was examined every hour using MALDI-TOF MS (see Methods) in the same stock suspension of fecal inoculum.

The qualitative microbiological analysis demonstrated a significant decrease in the number of bacterial strains after only 10 h of incubation at 37 °C because of the log phase of microbial competition for population growth (Fig S1).


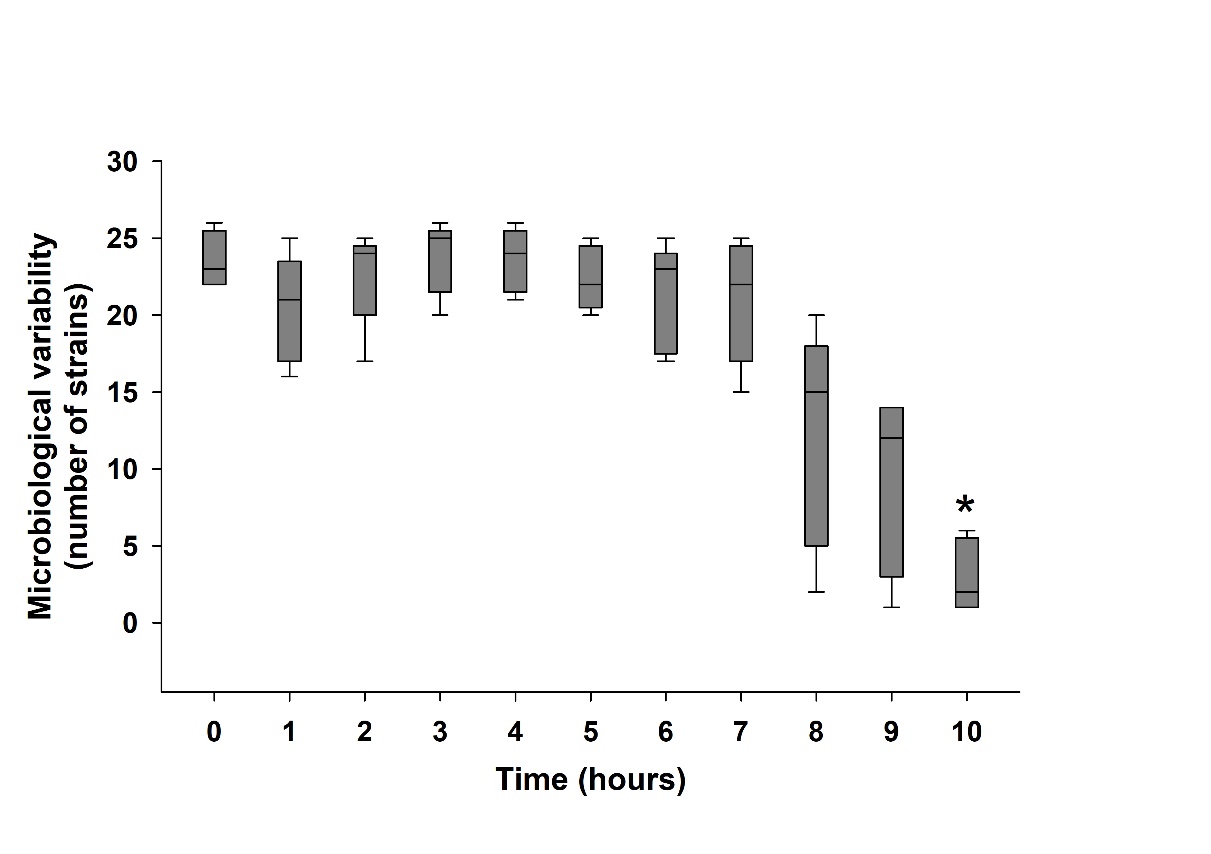


**Supplementary Figure S1. Microbiological diversity in the fecal inoculum as a function of incubation time*.*** Plots demonstrate the median value (horizontal line in the box) and the 25^th^ (lower whisker) and 75^th^ (upper whisker) percentiles (n = 4–5 at each time point). Between-group analysis: Kruskal–Wallis test and Dunn’s post-hoc test. ^*^*P* < 0.05 vs microbial diversity of the initial suspension).

## S2.2. The effects of 12 h storage time at 4 °C on changes in germ count and composition of the induction inoculum

**Supplementary Table S2. Effect of 12 h storage at 4 °C on the concentration and composition of the inducer inoculum in representative samples (#1–5);** Differences in CFUs of inocula between 0 h and 12 h were analyzed with the Wilcoxon test.

| **No.** | **0 h** | | **12 h** | |  |
| --- | --- | --- | --- | --- | --- |
|  | **CFU/mL** | **Microbial composition** | **CFU/mL** | **Microbial composition** |  |
| **1** | 2.40×10^8^ | *E. coli, Lactobacillus johnsonii, Lactobacillus murinus, Bacteroides thetaiotaomicron, Pseudomonas aeruginosa, Klebsiella pneumoniae* | 1.97×10^8^ | *E. coli, Lactobacillus johnsonii, Lactobacillus murinus, Bacteroides thetaiotaomicron, Pseudomonas aeruginosa, Klebsiella pneumoniae* |  |
| **2** | 6.66×10^7^ | *E. coli, Lactobacillus murinus, Lactobacillus garvieae, Micrococcus luteus, Klebsiella pneumoniae* | 6.62×10^7^ | *E. coli, Lactobacillus murinus, Lactobacillus garvieae, Micrococcus luteus, Klebsiella pneumoniae* |  |
| **3** | 1.0×10^7^ | *E. coli, Lactobacillus murinus, Lactobacillus johnsonii, Klebsiella pneumoniae* | 9.97×10^6^ | *E. coli, Lactobacillus murinus, Lactobacillus johnsonii, Klebsiella pneumoniae* |  |
|  |  |  |  |  |  |
| **4** | 1.07×10^7^ | *E. coli, Bacteroides xylanisolvens, Klebsiella pneumoniae* | 1.04×10^7^ | *E. coli, Bacteroides xylanisolvens, Klebsiella pneumoniae* |  |
| **5** | 2.04×10^6^ | *E.coli, Lactobacillus murinus, Pseudomonas mucidolens, Lactobacillus rhamnosus* | 2.11×10^6^ | *E.coli, Lactobacillus murinus, Pseudomonas mucidolens, Lactobacillus rhamnosus* |  |
| **Median** | 1.07×10^7^ | | 1.04×10^7^ (*P* = 0.094) | |  |

A statistically non-significant (3%) decrease was detected during the 12 h storage time of the fecal suspension. There was no change in composition.

# Supplementary Table S3. Assessment scheme of the condition of animals with rat sickness scores.

| Criteria | | | Time (h) | | | | | | |
| --- | --- | --- | --- | --- | --- | --- | --- | --- | --- |
|  |  |  | 0 | 6 | 12 | 24 | 36 | 48 | 72 |
| Condition of fur | 0 | Not altered |  |  |  |  |  |  |  |
|  | 1 | Piloerection |  |  |  |  |  |  |  |
| Posture | 0 | Not altered |  |  |  |  |  |  |  |
|  | 1 | Altered weight distribution |  |  |  |  |  |  |  |
|  | 2 | Hunched back |  |  |  |  |  |  |  |
| Mobility | 0 | Not altered |  |  |  |  |  |  |  |
|  | 1 | Slow/stiff movement |  |  |  |  |  |  |  |
|  | 2 | No movement when disturbed |  |  |  |  |  |  |  |
| Alertness | 0 | Not altered |  |  |  |  |  |  |  |
|  | 1 | Decreased |  |  |  |  |  |  |  |
|  | 2 | No reaction |  |  |  |  |  |  |  |
| Weight | 0 | +0-5-10 g |  |  |  |  |  |  |  |
|  | 1 | -5-10 g |  |  |  |  |  |  |  |
|  | 2 | -20 g |  |  |  |  |  |  |  |
| Temperature | 0 | Not altered |  |  |  |  |  |  |  |
|  | 1 | Fever or hypothermia |  |  |  |  |  |  |  |
| **TOTAL SCORE** for each animal at the indicated time points | | |  |  |  |  |  |  |  |
